# Hepatitis C Virus Core-Derived Peptides Inhibit Genotype 1b Viral Genome Replication via Interaction with DDX3X

**DOI:** 10.1371/journal.pone.0012826

**Published:** 2010-09-17

**Authors:** Chaomin Sun, Cara T. Pager, Guangxiang Luo, Peter Sarnow, Jamie H. D. Cate

**Affiliations:** 1 California Institute for Quantitative Biosciences, University of California, Berkeley, California, United States of America; 2 Department of Microbiology and Immunology, Stanford University School of Medicine, Stanford, California, United States of America; 3 Department of Microbiology, Immunology and Molecular Genetics, University of Kentucky, Lexington, Kentucky, United States of America; 4 Department of Chemistry, University of California, Berkeley, California, United States of America; 5 Department of Molecular and Cell Biology, University of California, Berkeley, California, United States of America; 6 Physical Biosciences Division, Lawrence Berkeley National Laboratory, Berkeley, California, United States of America; Yonsei University, Republic of Korea

## Abstract

The protein DDX3X is a DEAD-box RNA helicase that is essential for the hepatitis C virus (HCV) life cycle. The HCV core protein has been shown to bind to DDX3X both *in vitro* and *in vivo*. However, the specific interactions between these two proteins and the functional importance of these interactions for the HCV viral life cycle remain unclear. We show that amino acids 16–36 near the N-terminus of the HCV core protein interact specifically with DDX3X both *in vitro* and *in vivo*. Replication of HCV replicon NNeo/C-5B RNA (genotype 1b) is significantly suppressed in HuH-7-derived cells expressing green fluorescent protein (GFP) fusions to HCV core protein residues 16–36, but not by GFP fusions to core protein residues 16–35 or 16–34. Notably, the inhibition of HCV replication due to expression of the GFP fusion to HCV core protein residues 16–36 can be reversed by overexpression of DDX3X. These results suggest that the protein interface on DDX3X that binds the HCV core protein is important for replicon maintenance. However, infection of HuH-7 cells by HCV viruses of genotype 2a (JFH1) was not affected by expression of the GFP fusion protein. These results suggest that the role of DDX3X in HCV infection involves aspects of the viral life cycle that vary in importance between HCV genotypes.

## Introduction

Hepatitis C virus (HCV) infection is a major cause of chronic hepatitis, liver cirrhosis and hepatocellular carcinoma worldwide [1]. HCV is a single-strand RNA virus whose 9.6 kb genome contains a single large translational open reading frame that encodes a large polyprotein precursor of 3010–3033 amino acids [2]. HCV core protein, the first structural protein encoded by the HCV open reading frame, is a highly conserved RNA-binding protein that forms the viral nucleocapsid [3].

To date, approximately 180 million people are infected with HCV worldwide [4]. However, a protective vaccine is not yet available and therapeutic options are still limited [1]. Thus, development of new classes of antiviral compounds is urgently needed. In the past few years, many inhibitors targeting the viral proteins have become available [5], [6]. However, for these kinds of inhibitors, resistance to treatment will arise quickly over time, as observed with, for example, HIV or HBV patients during therapy [7]. On the other hand, targeting a host factor rather than a viral protein might make it more difficult for a virus to develop resistance against the drug [8].

DDX3X is a human RNA helicase that is ubiquitously expressed in a wide range of tissues [9]. DDX3X has been implicated in several cellular functions, including splicing [10], [11], translation initiation and repression [12], [13], cell cycle regulation [14], [15], [16], [17], nucleo-cytoplasmic RNA shuttling [18], RNA transport [19], interferon induction [20], [21] and apoptosis [22]. Two separate studies recently provided evidence for the involvement of DDX3X in HCV replication [23], [24]. Due to the finding that HIV and HCV seem to require DDX3X for their replication [18], [23], the inhibition of DDX3X may serve as a novel therapeutic strategy for the development of drugs against these viruses [7].

Several reports show direct interaction between HCV core protein and DDX3X, in both cytoplasmic and nuclear compartments [25], [26], [27]. In HCV infected cell lines, DDX3X localization shifts from the nucleus to the cytoplasm, where it concentrates near the endoplasmic reticulum in discrete foci. Interestingly, DDX3X also co-localized with HCV core protein [26]. However, the importance for a direct interaction between DDX3X and HCV core has been questioned [28], and the exact role for this interaction and co-localization have not been determined to date. DDX3X can interact with the muti-component translation initiation factor eIF3 indicating that DDX3X may play a role in translation initiation [29]. Furthermore, eIF3 interacts with the HCV internal ribosome entry site (IRES) in the 5′-untranslated region of the genomic RNA [30], suggesting that DDX3X may be involved in the translation of the HCV polyprotein.

In order to determine the involvement of DDX3X in the HCV viral life cycle, protein fusions to HCV core protein regions were isolated that interact with DDX3X both *in vitro* and *in vivo*. These fusion proteins were used as possible decoys to compete with interactions between DDX3X and viral and cellular factors in cell-based assays. We tested whether this kind of competition would be sufficient to suppress maintenance of the HCV replicon or HCV viral infectivity in HuH-7 cells.

## Materials and Methods

### Plasmids

A PCR product encoding the cytoplasmic domain of HCV core protein (amino acids 1–115) was cloned into pGEX2T (Amersham Pharmacia) to construct pGEXHCVc1-115, which encodes glutathione-S-transferase (GST) fused in frame N-terminally to HCV core protein residues 1–115. The plasmids expressing related GST fusion proteins to HCV core protein segments were constructed by using Quikchange mutagenesis (Stratagene). The helicase domain of human protein DDX3X (amino acids 168–582, based on DDX3X NCBI reference sequence NP_001347.3) was cloned into pET23a (Novagen) to generate a vector encoding the DDX3X helicase domain with an N-terminal His_6_ tag. HCV core protein residues were also cloned into the vector pmaxGFP (Amaxa) to generate N-terminal GFP fusions to truncated HCV core proteins (Table 1). In addition, the mCherry fluorescent protein was fused to the C-terminus of DDX3X in place of GFP, using the same plasmid background. The resulting mammalian expression vectors utilize the CMV transcription promoter. All plasmid sequences were confirmed by DNA sequencing.

**Table 1:** Plasmids used in this study.

Plasmids	Description	Reference or source
pGEX2T	4.97 kb, IPTG-inducible expression vector for GST fusion	Amersham Pharmacia
pGEXHCVc1-115	pGEX2T derived expression plasmid for HCV core (aa 1–115)	This study
pGEXHCVc1-34	pGEX2T derived expression plasmid for HCV core (aa 1–34)	This study
pGEXHCVc1-35	pGEX2T derived expression plasmid for HCV core (aa 1–35)	This study
pGEXHCVc1-36	pGEX2T derived expression plasmid for HCV core (aa 1–36)	This study
pGEXHCVc11-36	pGEX2T derived expression plasmid for HCV core (aa 11–36)	This study
pGEXHCVc16-34	pGEX2T derived expression plasmid for HCV core (aa 16–34)	This study
pGEXHCVc16-35	pGEX2T derived expression plasmid for HCV core (aa 16–35)	This study
pGEXHCVc16-36	pGEX2T derived expression plasmid for HCV core (aa 16–36)	This study
pGEXHCVc21-36	pGEX2T derived expression plasmid for HCV core (aa 21–36)	This study
pET23a	3.66 kb IPTG-inducible expression vector with His_6_ tag	Novagen
pETDDX3X168-582	pET23a derived expression plasmid for DDX3X (aa 168–582)	This study
pmaxGFP	3.48 kb mammalian GFP expression vector	Amaxa
pGFPHCVc16-34	pmaxGFP derived expression plasmid for HCV core (aa 16–34)	This study
pGFPHCVc16-35	pmaxGFP derived expression plasmid for HCV core (aa 16–35)	This study
pGFPHCVc16-36	pmaxGFP derived expression plasmid for HCV core (aa 16–36)	This study
pmCherry-N1	4.72 kb mCherry expression vector for C-terminal fusions	Clontech
pDDX3XmCherry	Expression plasmid for mCherry C-terminal fusion to full length DDX3X	This study
pcDNA3	5.4 kb mammalian expression vector	Invitrogen
pDiLuc	pcDNA3 derived plasmid expressing cap-dependent Renilla luciferase and HCV IRES-dependent Firefly luciferase	This study

### *In vitro* interaction of HCV core protein fragments with DDX3X

Interactions between the DDX3X helicase domain and HCV core protein fusion peptides were assayed using GST pull-downs (ProFound, Pierce). Briefly, *E. coli* BL21 cells carrying expression plasmids (Table 1) were treated with 0.3 mM isopropyl-β-D-thiogalactopyranoside (IPTG) to induce fusion protein expression. The bacterial cells were harvested, pelleted, and resuspended in 1 mL of phosphate buffered saline (PBS) per 5 mL of bacterial culture. The cells were then disrupted by sonication on ice. Triton X-100 was added to a final concentration of 0.01%, and proteins were purified using glutathione-Sepharose resin (GE Healthcare). Purified GST fusion proteins (1 µM in 1 mL) were mixed gently with 0.1 mL reduced glutathione-Sepharose 4B beads (GE Healthcare) for 60 min at 4°C. The beads were collected by brief centrifugation and washed three times with PBS containing 0.01% Triton X-100. Purified DDX3X helicase domain (3 µM) was added to the beads in a volume of 1 mL and incubated for 3 h at 20°C with gentle rotation. The beads were washed 3 times with PBS containing 0.01% Triton X-100 per wash. Proteins bound to the beads were eluted in sodium dodecyl sulfate-polyacrylamide (SDS-PAGE) sample buffer, fractionated by SDS-PAGE (10% polyacrylamide gel) and stained by Coomassie blue.

### *In vitro* binding of GST-HCV core fusion proteins to endogenous DDX3X

HCV core protein fragments expressed as GST fusions from the pGST expression vectors (Table 1) were purified as described above. For *in vitro* binding assays, 0.1 mL of glutathione-Sepharose 4B beads (GE Healthcare) containing various GST fusion proteins (10 µg) were incubated with HuH-7 cell extracts (500 µg) at 4°C overnight with gentle rotation [27]. The beads were washed four times with 1 mL of PBS containing 0.01% Triton X-100 per wash. Proteins bound to the beads were eluted by SDS-PAGE sample buffer, fractionated by SDS-PAGE (10% polyacrylamide gel) and processed for Western blot analysis. The separated proteins were then electroblotted onto PVDF Western blotting membrane (Roche). After blocking for 1 h with 5% nonfat milk, the membranes were probed with primary antibody, washed with TBST (125 mM NaCl; 25 mM Tris pH 8.0; 0.1% Tween-20) 3 times, 10 min/per wash, and then incubated with horse radish peroxidase (HRP) labeled IgG (ProSci Incorporated, USA; 1∶5,000 final dilution). Unbound antibody was removed by washing and the blots were developed using a LumiBlot detection kit (Novagen, USA). Detection of DDX3X was performed with rabbit anti-DDX3X antiserum (ProSci Incorporated; 1∶1,000).

### Co-immunoprecipitation

Immunoprecipitation was performed essentially as described [31], with minor modifications. After transient transfection of HuH-7 cells (described below), the growth medium was removed and the cells were rinsed twice in cold phosphate-buffered saline, incubated for 30 min at 4°C in lysis buffer (50 mM Tris-HCl, pH 7.5, 150 mM NaCl, 1 mM EDTA, 1 mM dithiothreitol, 0.2 mM phenylmethylsulfonyl fluoride, and 1% NP-40), and collected by scraping. Cell debris was removed by centrifugation at 10,000×*g* for 10 min at 4°C. Extracts were precleared with protein G-agarose beads (Sigma) for 1 h at 4°C. The primary antibody of GFP (Evrogen, 1∶30,000) was added for 1 h at 4°C, and immunoglobulin complexes were incubated with protein G-agarose beads for 1 h at 4°C. The beads were extensively washed and analyzed by immunoblotting using the specific antibody against DDX3X as described above. For the detection of the GFP or GFP fusion HCV core proteins, rabbit anti-GFP antibody (Evrogen; 1∶30,000) was used.

### Cell culture, transfection and reporter assays

For HCV replication studies, a stable HuH-7 cell line containing HCV genotype 1b strain N genomic replicon NNeo/C-5B (hereafter named cell line 5B) was used [32]. The cell line was routinely grown in DMEM media supplemented with nonessential amino acids, penicillin, streptomycin, and 10% FBS (Omega Scientific) as described previously [32]. As indicated, 500 µg/mL G418-active ingredient (Geneticin, GIBCO Invitrogen) was added into the medium.

For inhibitory assays, cell line 5B was transfected with plasmids encoding GFP alone or encoding N-terminal GFP fusions to HCV core peptides using FuGENE 6 (Roche). Briefly, about 3×10^5^ 5B cells/10 mL medium were plated into each 10 cm plate 1 day prior to transfection. For each transfection, up to 6 µg of DNA was mixed with 18 µL of FuGENE 6 reagent diluted in 600 µL of Optimem media (Gibco BRL) and incubated for 20 min at room temperature. The DNA–FuGENE solution was then added directly to the cells. After 48 h, the cells were collected and split into two parts, one part for Northern blotting and the other for Western blot analysis. Transfection efficiency was assayed using FACS analysis in a C6 Flow Cytometer (Accuri Cytometers Inc., USA), and varied between 70%–100% (Figure S1).

For DDX3X overexpression assays, 3 µg of plasmids encoding GFP or GFP fusions to HCV core peptides (pmaxGFP, pGFPHCVc16-35, pGFPHCVc16-36) and 3 µg of plasmids encoding mCherry (pmCherry) or mCherry fusions to DDX3X (pDDX3XmCherry) were co-transfected into 3×10^5^ 5B cells. Forty-eight hours after transfection, the cells were collected and split into two parts, one part for Northern blotting and the other for Western blot analysis.

For the luciferase activity inhibitory assays, 3 µg of plasmids encoding GFP or GFP fusions to HCV core peptides (pmaxGFP, pGFPHCVc16-35, pGFPHCVc16-36) and 1 µg plasmid pDiluc encoding the cap-dependent Renilla luciferase and HCV IRES-dependent Firefly luciferase were co-transfected into 3×10^5^ 5B cells. Forty-eight hours after transfection, the cells were collected and split into three parts, one part for Northern blotting, the second for Western blotting and the third for luciferase activity analysis. A Dual-luciferase reporter system was used for luciferase activity analysis (Promega). In DDX3X rescue experiments, 3 µg of plasmid encoding mCherry or DDX3X-mCherry fusions (pmCherry-N1 or pDDX3XmCherry) were also included in the co-transfections.

### Western blot analysis of fusion protein expression in transiently transfected 5B cells

HuH-7 cells harboring the HCV replicon transiently transfected with plasmids pGFPHCVc16-35 or pGFPHCVc16-36, as described above, were assayed for HCV core peptide fusion protein expression by Western blot. The DDX3X and mCherry fusions to DDX3X were detected by a rabbit anti-DDX3X antibody (ProSci Incorporated; 1∶1,000). The rabbit anti-actin antibody (Sigma; 1∶1,000) was used to detect actin as the loading control.

### Northern blot analysis

Total cellular RNAs were extracted from HuH-7 cells by using the TRIzol reagent (Gibco-BRL) and were quantified using a Nanodrop spectrophotometer (Thermo Scientific) at 260 nm. The resulting total cellular RNA samples (10 µg) were denatured and fractionated by agarose gel [33]. The RNA was transferred from the gel to a nylon membrane by using 10 x SSC (1.5 M NaCl, 0.15 M Sodium Citrate), and immobilized on the membrane by UV cross-linking (Stratagene).

For monitoring HCV replicon expression, β-actin mRNA levels were used as an internal control in the Northern blot analyses as described [34]. DNA probes specific to the HCV replicon RNA and β-actin were [^32^P]-labeled using a RadPrime DNA labeling system (Invitrogen). For the detection of mRNA transcript integrity for luciferase assays, a probe spanning the 3′ 120 nuclueotides of the Renilla luciferase open reading frame (ORF) through the 5′ 126 nucleotides of the Firefly luciferase ORF was labeled as described above. Hybridization was carried out in ExpressHyb solution (CloneTech) for 1 h at 65°C. The membrane was washed 3×10 min at 55°C with 0.1 x SSC/0.1% SDS. Band intensities on the probed membranes were quantified by STORM phosphorimager analysis (Molecular Dynamics).

### JFH1 infection and pGFP-Core transfection of HuH-7 cells

Two protocols were applied to examine the effect of GFP-HCV core expression on JFH1 infection. In protocol one, HuH-7 cells were seeded in 6 cm culture plates and then transfected with 4 µg total plasmid DNA and FuGENE6, according to the manufacturer's specifications. Specifically, 0.25 µg pmaxGFP, 1 µg pGFPHCVc16-34, 4 µg pGFPHCVc16-35, and 2 µg pGFPHCVc16-36 and varying amounts of pcDNA3 (to yield 4 µg DNA) were transfected. Twenty-four hours after transfection, cells were infected with JFH1 virus for 5 h at 37°C. Infected cells were then trypsinized and re-seeded into two 10 cm culture plates. Protein and RNA extracts were harvested three days after infection. In protocol two, HuH-7 cells were seeded in a 10 cm culture dish and infected with JFH1 virus for 5 h at 37°C, trypsinized and re-plated into five 10 cm culture dishes. Two days after infection, cells were transfected with 8 µg total plasmid DNA via FuGENE6. Amounts of plasmid DNA transfected were as follows: 0.5 µg pmaxGFP, 2 µg pGFPHCVc16-34, 8 µg pGFPHCVc16-35, and 4 µg pGFPHCVc16-36 and varying amounts of pcDNA3 (to yield a total of 8 µg DNA). Cells were harvested 24 h later and levels of protein and RNA were analyzed by Western and Northern blot, respectively.

## Results

### HCV core peptides interact specifically with DDX3X *in vitro* and *in vivo*

**Figure 1:**
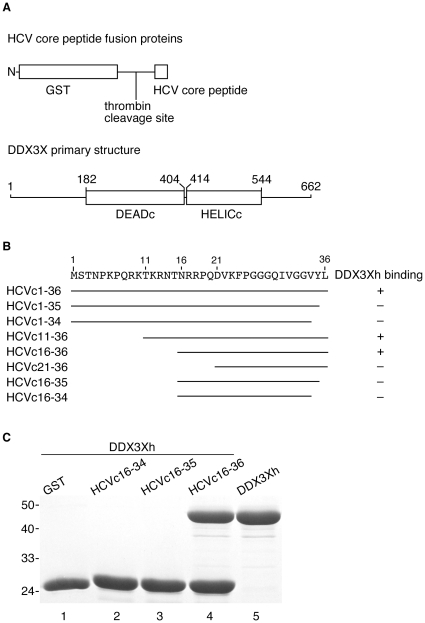
Pull-down assays of HCV core derived peptides and DDX3X helicase domain (DDX3Xh). (**A**). Schematic of the different GST fusions to the N-terminal fragments of the HCV core protein. Also shown is a schematic of DDX3X, with the DEADc and HELICc domains of the DEAD-box helicase labeled. (**B**). Schematic diagram of the regions of HCV core protein interacting with the DDX3X helicase domain (DDX3Xh) in pull-down assays. (**C**). Analysis of the interaction between DDX3Xh, GST or various GST fusions to HCV core proteins. Glutathione beads were used to pull down bound proteins prior to SDS gel electrophoresis. Lanes 1–4, GST fusions to the denoted HCV peptides incubated with DDX3Xh. Lane 5, DDX3Xh marker. Molecular weight markers are shown to the left, in kDa.

**Figure 2:**
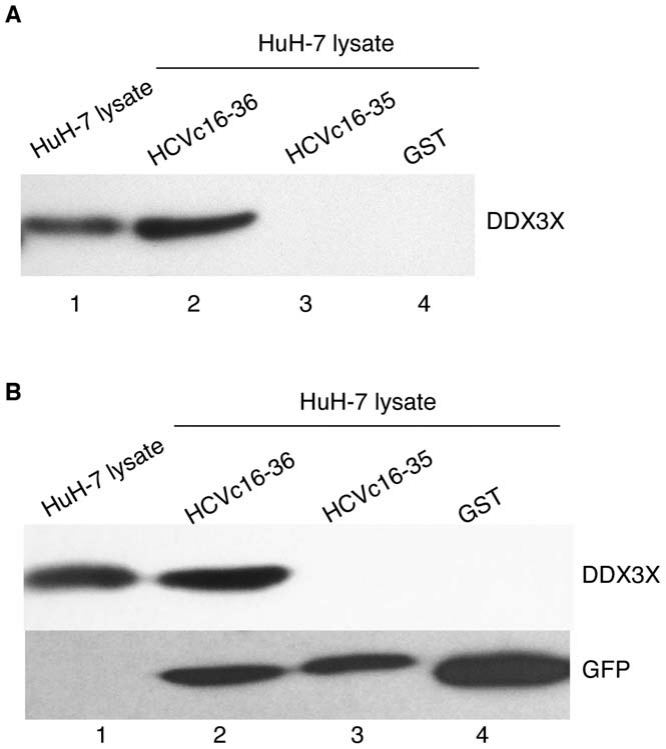
Interaction of DDX3X with HCVc16-36 *in vitro* and *in vivo*. (**A**). *In vitro* binding analysis of endogenous DDX3X and the various truncated forms of HCV core proteins. Western blot of GST or GST fusions to the denoted HCV core peptides incubated in HuH-7 cell lysate, pelleted using glutathione beads, and resolved by SDS gel electrophoresis. Lane 1, HuH-7 cell lysate input. Lanes 2–4, GST pull-down experiments. (**B**). Co-immunoprecipitation of GFP fusions to HCV core peptides and DDX3X from co-transfected HuH-7 cells. Immunoprecipitates were analysed by sequential immunoblotting with anti-DDX3X and anti-GFP antibodies. Lane 1, DDX3X marker from HuH-7 cell lysate. Lanes 2–4, co-immunoprecipitations of GFP fusions to the denoted HCV peptides using anti-GFP antibodies.

The domain of HCV core that interacts with DDX3X was originally mapped to the N-terminal 40 amino acids of the HCV core protein [27]. To determine the minimal requirements for specific HCV core peptide binding to DDX3X, a series of N-terminal GST fusions to HCV core peptides were used for pull-down assays (Figure 1B). The results showed that amino acids 1–15 (Figure 1B) and 37–40 (data not shown) were not necessary for the interaction with DDX3X helicase domain. Thus, HCV core peptides including amino acids 16 to 36 are sufficient for binding to the DDX3X helicase domain (Figure 1B, C). In contrast, HCV core peptides that include even one less residue, i.e. amino acids 16–35, did not bind to the DDX3X helicase domain (Figure 1B, C). Notably, the backbone of position 36, but not the sequence, is essential for binding of the core peptide to DDX3X (Figure S2)[28]. Additionally, when the GST-HCV core fusion peptides were used for pull-down assays with endogenous DDX3X from HuH-7 cells, the same specificity of binding was observed (Figure 2A).

To determine whether peptides from HCV core protein containing amino acids 16–36 are capable of binding to DDX3X in cells, HuH-7 cells were transiently transfected with vectors that express GFP or GFP fusions to HCV core peptides from amino acids 16–36 or 16–35. When cell lysates from transfected HuH-7 cells were immunoprecipitated by an anti-GFP antibody, Western blots revealed that DDX3X co-immunoprecipitated with the GFP fusion to HCV core peptide residues 16–36 (Figure 2B). In contrast, GFP fusions to HCV core peptides containing residues 16–35 did not co-immunoprecipitate with DDX3X (Figure 2B). These results indicate that the HCV core peptide containing residues 16–36 bound specifically to DDX3X in HuH-7 cells, consistent with the *in vitro* assays with purified proteins.

### HCVc16-36 inhibits the replication of a genotype 1b-derived HCV replicon

**Figure 3:**
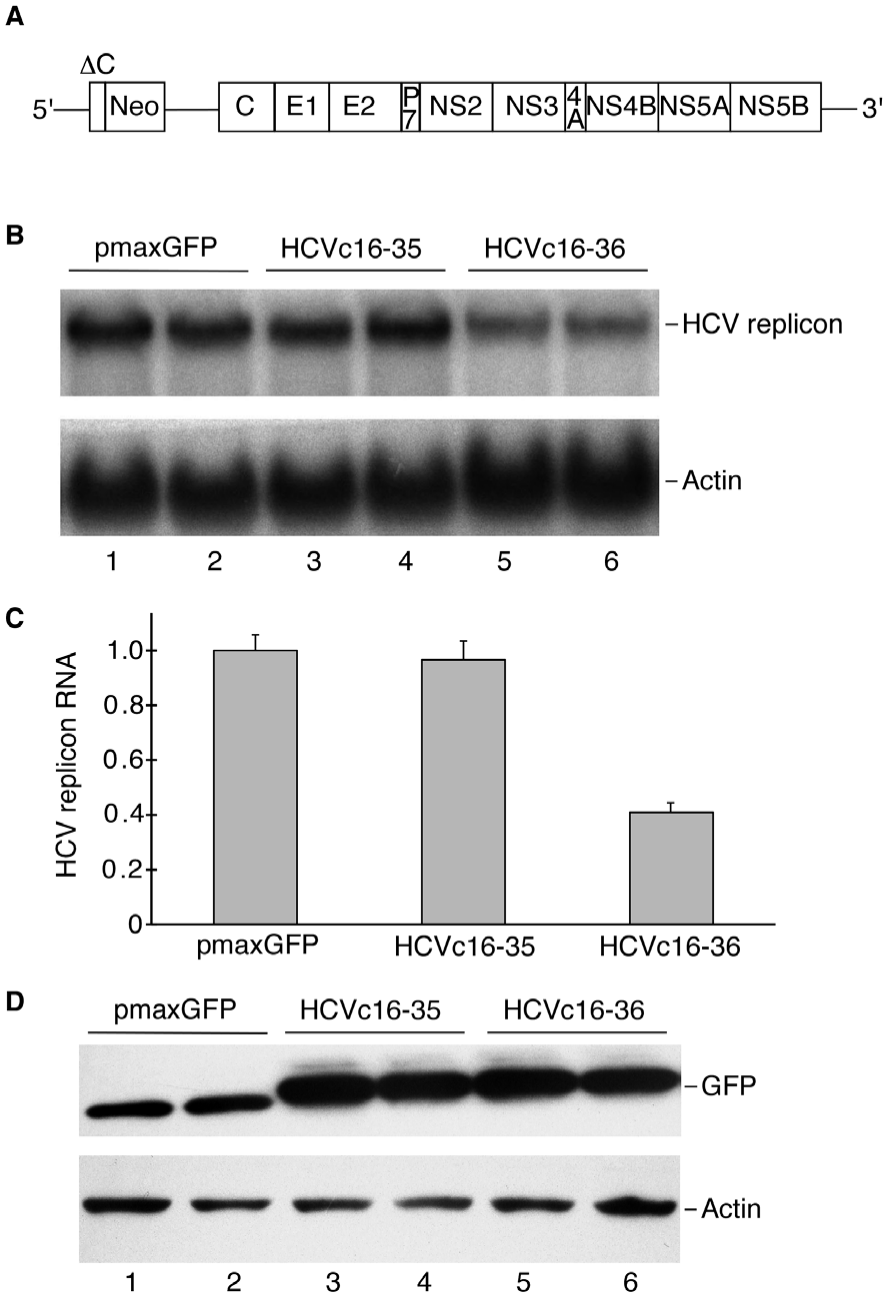
Effects of HCVc16-36 on HCV replicon RNA abundance. (**A**). Diagram of the HCV replicon NNeo/C-5B (30) used in the experiment. The plasmid encodes the 5′-UTR of genotype 1b HCV-N immediately upstream of the sequence encoding the N-terminal 12 amino acids of the core protein fused in-frame to the selectable marker, Neo. The Neo gene is followed by the IRES of EMCV fused to the full genotype 1b HCV-N polyprotein sequence and 3′-UTR (30). (**B**). Northern blot analysis of HCV replicon RNA remaining in HuH-7 cells 48 h after transient transfection with GFP fusion protein encoding plasmids: pmaxGFP, pGFPHCVc13-35 (HCVc16-35), and pGFPHCVc16-36 (HCVc16-36). Northern blots of actin mRNA levels serve as a loading control. Duplicate experiments are shown in lanes 12, 34, and 56. (**C**). Quantification of HCV replicon RNA from panel B. Experiments were carried out in duplicate and columns represent the quantity of HCV RNA, normalized to β-actin mRNA, and to the levels in pmaxGFP transfected cells. Error bars represent the standard deviation from the mean for the experiments. (**D**). Expression of GFP fusions to N-terminal fragments of the HCV core protein. HuH-7 cells were transiently transfected with plasmids encoding the GFP fusions, and the fusion proteins were detected 48 h post-transfection by Western blotting with an anti-GFP antibody. Western blots of actin serve as loading controls.

Since HCV core peptides containing residues 16–36 are capable of binding DDX3X in cells, and given the importance of DDX3X for HCV viral infectivity [23], [24], it is possible that expression of these HCV core peptides might block DDX3X interactions with cellular or viral factors that are required during the viral life cycle. To test whether HCV core peptides could inhibit HCV replication, GFP fusions to HCV core peptides were transiently expressed in HuH-7 cells harboring the NNeo/C-5B HCV replicon derived from HCV genotype 1b (Figure 3A). When the HuH-7 cells were transiently transfected with plasmids encoding a GFP fusion to HCV core protein amino acids 16-36, the levels of the HCV replicon RNA dropped by 50% after 48 h (Figures 3B, C). Stable transfection of a plasmid encoding a GST fusion to HCV core protein amino acids 16–36 also resulted in very low levels of HCV replicon RNA after several days of cell culture (data not shown). Notably, no inhibition was observed when cells were transiently or stably transfected with plasmids that encoded a GFP or GST fusion to the fragment of the HCV core protein comprising amino acids 16–35 (Figures 3B, C).

**Figure 4:**
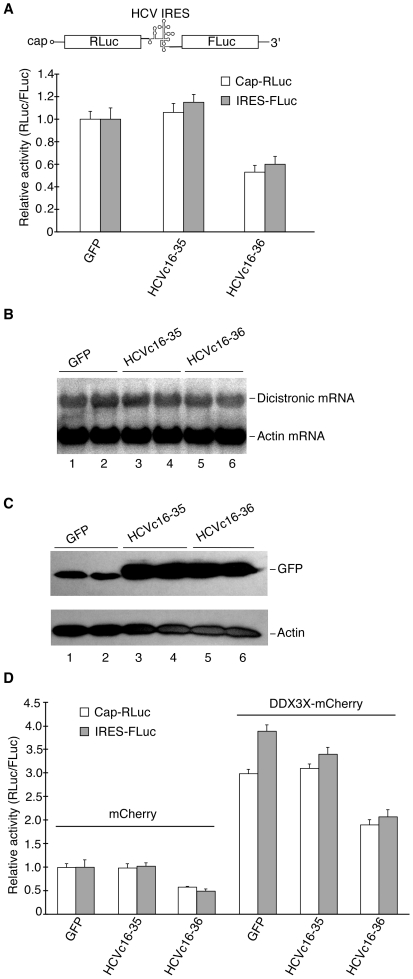
Inhibition of cap- and HCV IRES-dependent translation by HCVc16-36. (**A**). Schematic of the pDiLuc plasmid, and the effects of expression of GFP fusions to HCV core peptides on cap- and HCV IRES-dependent luciferase expression in 5B cells. RLuc, *Renilla* luciferase; FLuc, Firefly luciferase. (**B**). Northern blot analysis of the dicistronic luciferase mRNA from cells transfected with plasmids encoding GFP or GFP fusions to HCV core peptides (HCVc16-35 and HCVc16-36) and dicistronic luciferase vectors. (**C**). Western blot analysis of the expression of GFP fusions to HCV core peptides of the samples used in panels A and B. (**D**). Partial rescue of activities of cap- and HCV IRES-dependent luciferases repressed by the HCVc16-36. Co-expression of mCherry in controls is compared to co-expression of DDX3X-mCherry.

To determine the possible mechanism by which maintenance of the NNeo/C5-B replicon in HuH-7 cells was suppressed, HCV core peptide fusions were transiently transfected into HuH-7 cells expressing luciferase. The activities of cap-dependent and HCV IRES-dependent luciferases were repressed by the expression of GFPHCVc16-36 compared to the expression of GFP and GFPHCVc16-35 (Figure 4). The repression seemed to be at the level of translation but not at the level of transcription because overall reporter RNA abundance was not affected (Figure 4B). Notably, inhibition of luciferase expression by HCV core peptides could be rescued by overexpression of DDX3X (Figure 4D), supporting the hypothesis that HCVc16-36 interacts with DDX3X *in vivo*.

**Figure 5:**
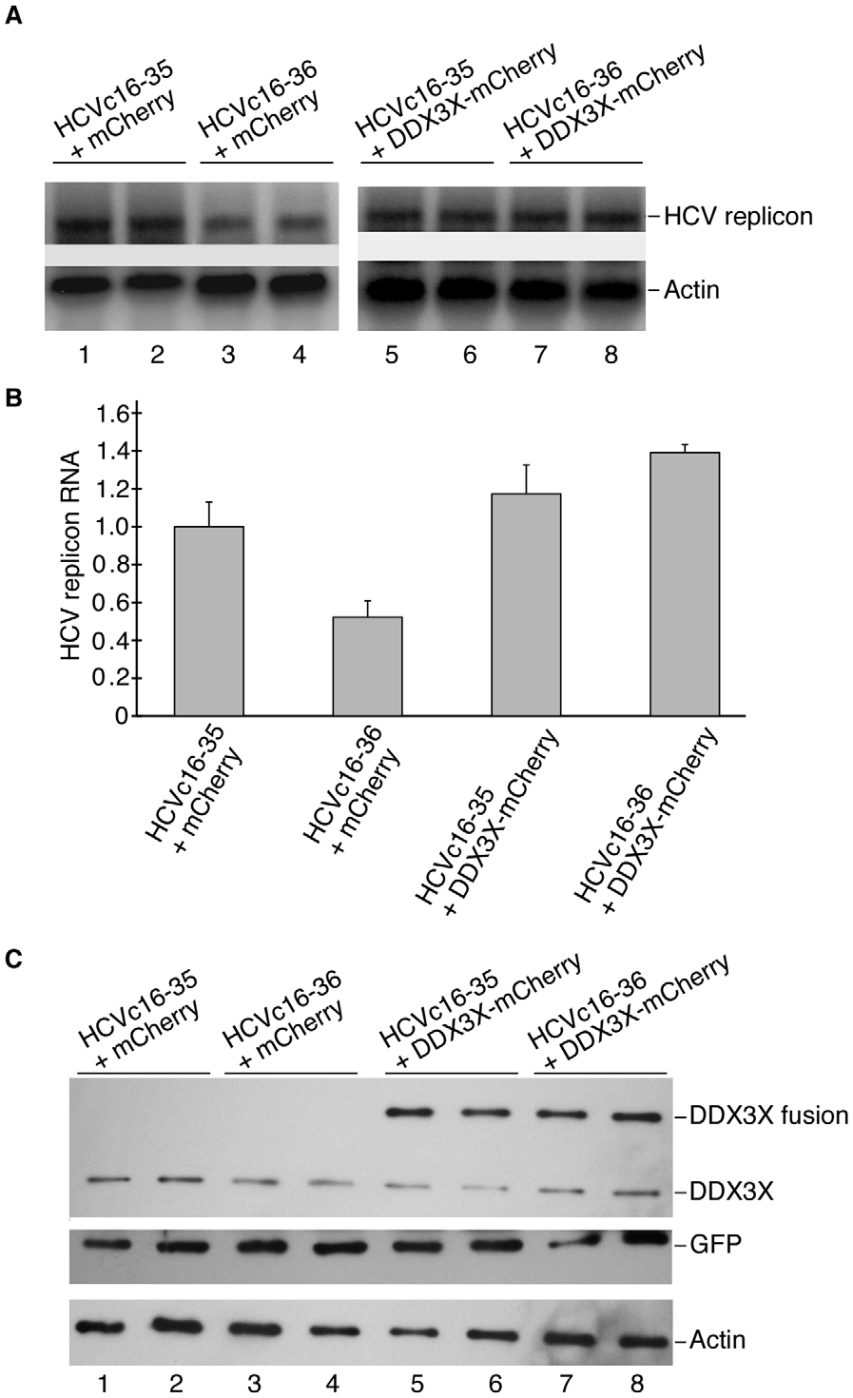
Rescue of HCV replicon RNA by DDX3X in HuH-7 cells. (**A**). Northern blot of HCV replicon RNA levels in cells expressing GFP fusions to HCV core peptides (HCVc16-35, HCVc16-36) in the absence (mCherry) or presence of exogenously expressed DDX3X fused to mCherry (DDX3X-mCherry). (**B**). Quantification of the HCV replicon RNA levels in panel A. Experiments were done in duplicate and columns represent the percentage of HCV replicon RNA normalized to β-actin mRNA levels. (**C**). Western blot analysis of the expression of DDX3X and GFP fusions to HCV core peptides in the samples used in panel A.

In order to examine whether the decrease in HCV replicon RNA is due to inhibition of endogenous DDX3X activity, vectors encoding the mCherry fusion to DDX3X as well as the GFP fusions to HCV core peptides were transfected into the HuH-7 cells harboring the NNeo/C-5B replicon. Expression of GFP fusions to HCV core peptide residues 16-36 reduced replicon RNA levels when mCherry alone was co-transfected (Figures 5A and B). However, the HCV core peptide fusion did not decrease viral RNA abundance when the cells expressed both the GFP-HCV core peptide fusion and mCherry fused to DDX3X (Figures 5A and B). In fact, the expression of exogenous DDX3X in the cells slightly increased the amount of the HCV replicon (Figure 5B).

### HCV core peptides do not inhibit HCV virus JFH1 infection of HuH-7 cells

**Figure 6:**
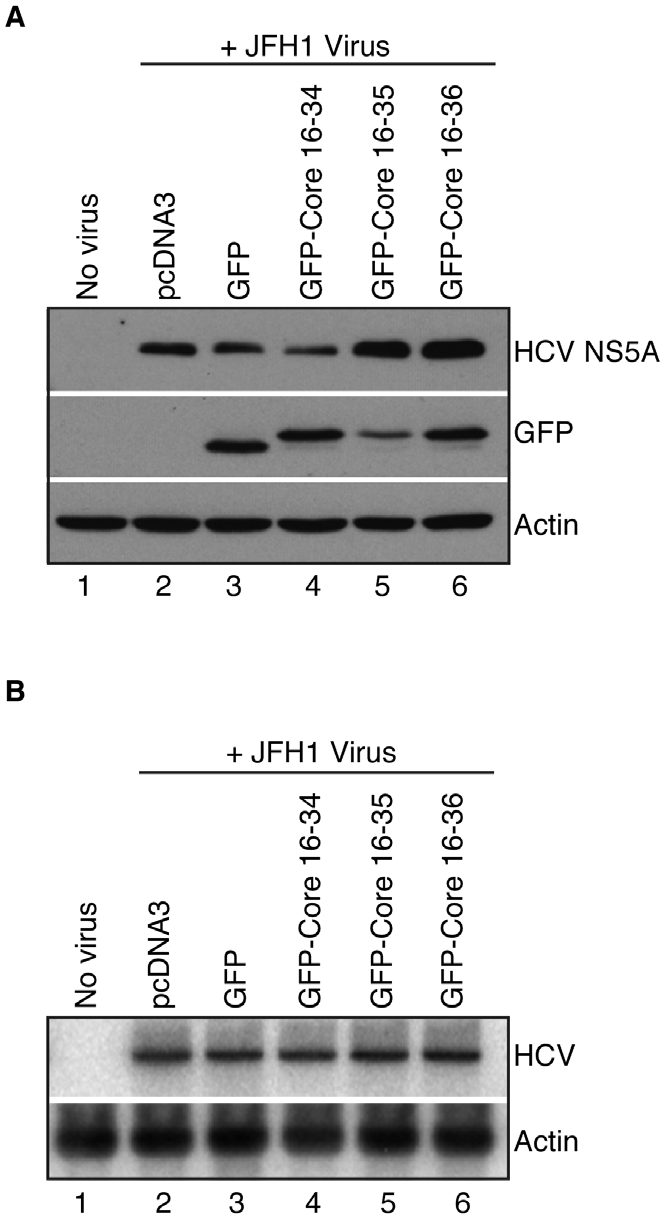
HCV protein and RNA abundances in HuH-7 cells expressing GFP-Core fusion proteins, following infection with JFH1 virus. HuH-7 cells were transfected with pGFP-Core plasmids (pGFPHCVc16-34, pGFPHCVc16-35, pGFPHCVc16-36), infected with JFH1 virus and harvested three days after infection. (**A**) HCV and GFP protein levels were examined in Western blots using anti-NS5A MAb 9E10, anti-GFP and anti-Actin antibodies. (**B**) HCV and actin RNA levels were analyzed in Northern blots.

**Figure 7:**
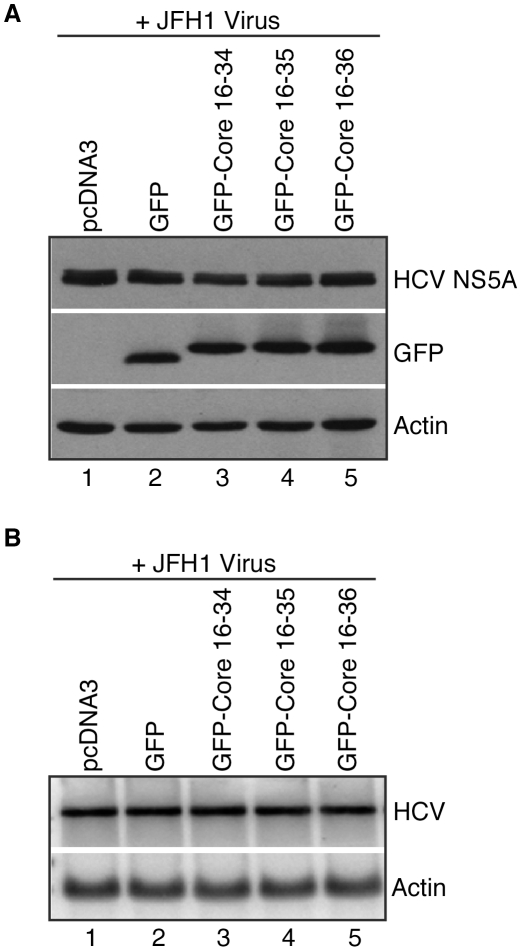
HCV protein and RNA abundances in HuH-7 cells after infection with JFH1 virus, followed by transfection of plasmids encoding GFP and GFP-Core peptides. HuH-7 cells were infected with JFH1 virus. Two days after infection cells were transfected with pGFP-Core plasmids (pGFPHCVc16-34, pGFPHCVc16-35, pGFPHCVc16-36). (**A**) HCV and GFP-Core fusion protein levels were examined in Western blots using anti-NS5A MAb 9E10, anti-GFP and anti-Actin antibodies. (**B**) HCV and actin RNA levels were analyzed in Northern blots.

HCV replicons recapitulate some but not all aspects of the HCV viral life cycle. In order to test whether the effect of HCV core peptide expression could decrease HCV viral infectivity more broadly, HuH-7 cells were assayed for JFH1 HCV infection in two different ways. In one experiment, HuH-7 cells were first transiently transfected with vectors encoding the GFP-HCV core protein fusions, and then infected with the JFH1 virus. Three days after infection, the cells were harvested and HCV viral RNA and NS5A protein levels were analyzed. In these experiments, expression of HCV peptides had no appreciable effect on the levels of HCV RNA and protein, although levels of GFP fusion protein expression were similar (Figure 6). In another experiment, HuH-7 cells were first infected with JFH1 virus. Two days after infection, the cells were transfected with vectors encoding the GFP fusions to HCV core peptides. As in the first experiment, expression of fusions to HCV core peptides had no discernible effect on HCV viral RNA or NS5A protein levels (Figure 7).

## Discussion

The high prevalence of disease caused by HCV and the limited efficacy of interferon-based therapies have stimulated the search for safer and more effective drugs to treat HCV infection [35]. In the past few years, more and more inhibitors targeting the viral protease and polymerase had been developed [5]. Recently, new compounds have been discovered that target HCV protein NS5A [6]. However, resistance to these kinds of inhibitors has been problematic. One possible solution to the buildup of resistance mutations in the virus is to target host cellular factors. Theoretically, a drug that targets a cellular factor that is important for the viral life cycle could generally inhibit all viruses that depend on the same host factor [7]. Given that DDX3X seems to be required for replication of HCV and HIV, it appears that DDX3X could be a promising target for drug development against these two viruses that pose major global health threats [7].

It was recently shown that DDX3X is required for HCV RNA replication, with infectious virus production strongly inhibited [24] and HCV replicon stability partially suppressed by DDX3X knock-downs [23]. These results indicate that DDX3X plays a crucial role in the HCV life cycle. HCV core protein interactions with DDX3X could be important for this effect. In order to disrupt possible interactions between DDX3X and its viral or host factors responsible for DDX3X stimulation of HCV propagation, we first mapped the minimal segment of HCV core protein that is capable of interacting with DDX3X. The interaction domain of HCV core to the DDX3X was originally mapped to the N-terminal 40 amino acids of the HCV core protein [27]. Based on this result, we mapped the minimal interaction region of HCV core, both *in vitro* and *in vivo*, to include amino acids 16 to 36 (Figure 1, Figure 2). We then overexpressed fusions to HCV core protein residues 16-36 to test their ability to suppress HCV replicon maintenance or HCV viral infectivity. These fusions did act as competitors for DDX3X by suppressing NNeo/C5-B replicon maintenance (Figure 3), which could be rescued by overexpression of DDX3X (Figure 5). Furthermore, suppression of HCV replicon maintenance by the fusions had the same core peptide length dependence seen for binding to DDX3X.

Interestingly, a recent report using a JFH1-based replicon showed that the interaction between the HCV core protein and DDX3X is not required for HCV replicon maintenance [28], seemingly in contradiction with our results with the NNeo/C-5B replicon. It is possible that HCV genotypic differences between JFH1 viruses (genotype 2a) and the NNeo/C-5B replicon (genotype 1b) are the reason for the differences seen here. Notably, the only sequence difference in the two genotypes within core protein residues 16-36 occurs at position 20, a position that is not important for interactions with DDX3X (Figure S3) [28]. It is possible that the potency of the peptides as competitors are not strong enough to be detected in the context of JFH1 viruses. It is known that JFH1 replicons exhibit a higher efficiency of HuH-7 colony formation when compared to genotype 1b (HCV-N-derived) replicons such as NNeo/C5-B, even though their replicating RNA copy numbers are similar [36]. Furthermore, JFH1–based replicons were found to be more resistant to interferon than HCV-N-based replicons such as NNeo/C5B [36]. We propose that the fusions to the HCV core peptides used in the present studies block a protein interaction interface on DDX3X that is important for binding viral or host factors involved in HCV replication. While it is probable that the HCVc16-36 core peptide competes with direct interactions between HCV core and DDX3X [25], [26], [27], interference with DDX3X binding to other viral or host factors could be more important [28].

In the present experiments, HCV core-derived peptides containing amino acids 16-36 can bind DDX3X and thereby inhibit HCV NNeo/C5-B replicon maintenance in HuH-7 cells. Thus, the HCV core peptide identified here could serve as a potential inhibitor of HCV that targets a cellular protein factor [37]. It will be interesting in future experiments to unravel the many potential functions of DDX3X that may be involved in the HCV viral life cycle [8], and how interactions of the HCV core peptides used here inhibit these functions of DDX3X. A long-range goal would be to improve the potency of competitors that bind to DDX3X and specifically block its function in the viral life cycle of multiple HCV genotypes.

## Supporting Information

Figure S1 FACS analysis of GFP-HCV core fusion transfection efficiency. Histograms of cell number versus GFP fluorescence are shown for an empty vector (pmaxGFP without *gfp* gene), GFP expression vector (pmaxGFP), or vectors expressing GFP fusions to HCV core peptides 16-35 (pGFPHCVc16-35) or 16-36 (pGFPHCVc16-36). Transfection efficiencies listed below each panel are estimated based on comparisons to the empty vector control.
(0.35 MB TIF)

Figure S2 Pull-down assays of HCV core derived peptides with mutations at position 36 and DDX3X helicase domain (DDX3Xh). Analysis of the interaction between DDX3Xh, GST or various GST fusions to HCV core proteins mutated at position 36. Glutathione beads were used to pull down bound proteins prior to SDS gel electrophoresis. Lanes 2–6, GST fusions to the denoted HCV peptides incubated with DDX3Xh. Lanes 1 and 7, GST and DDX3Xh markers, respectively. Molecular weight markers are shown to the left, in kDa.
(0.49 MB TIF)

Figure S3 Pull-down assays of HCV core derived peptides mutated at position 20 and DDX3X helicase domain (DDX3Xh). Analysis of the interaction between DDX3Xh, GST or GST fusions to HCV core peptides. Glutathione beads were used to pull down bound proteins prior to SDS gel electrophoresis. Lanes 2–3, GST fusion to the denoted HCV peptides incubated with DDX3Xh. Lanes 1 and 4, GST and DDX3Xh markers, respectively. All samples were resolved on the same SDS gel, with intervening lanes removed for clarity. Molecular weight markers are shown to the left, in kDa.
(0.18 MB TIF)
