# Supplementary figures and images for: Hepatitis C Virus Core-Derived Peptides Inhibit Genotype 1b Viral Genome Replication via Interaction with DDX3X
Source: PLoS One. 2010 Sep 17;5(9):e12826. doi: 10.1371/journal.pone.0012826 (PMC2941470; doi:10.1371/journal.pone.0012826)

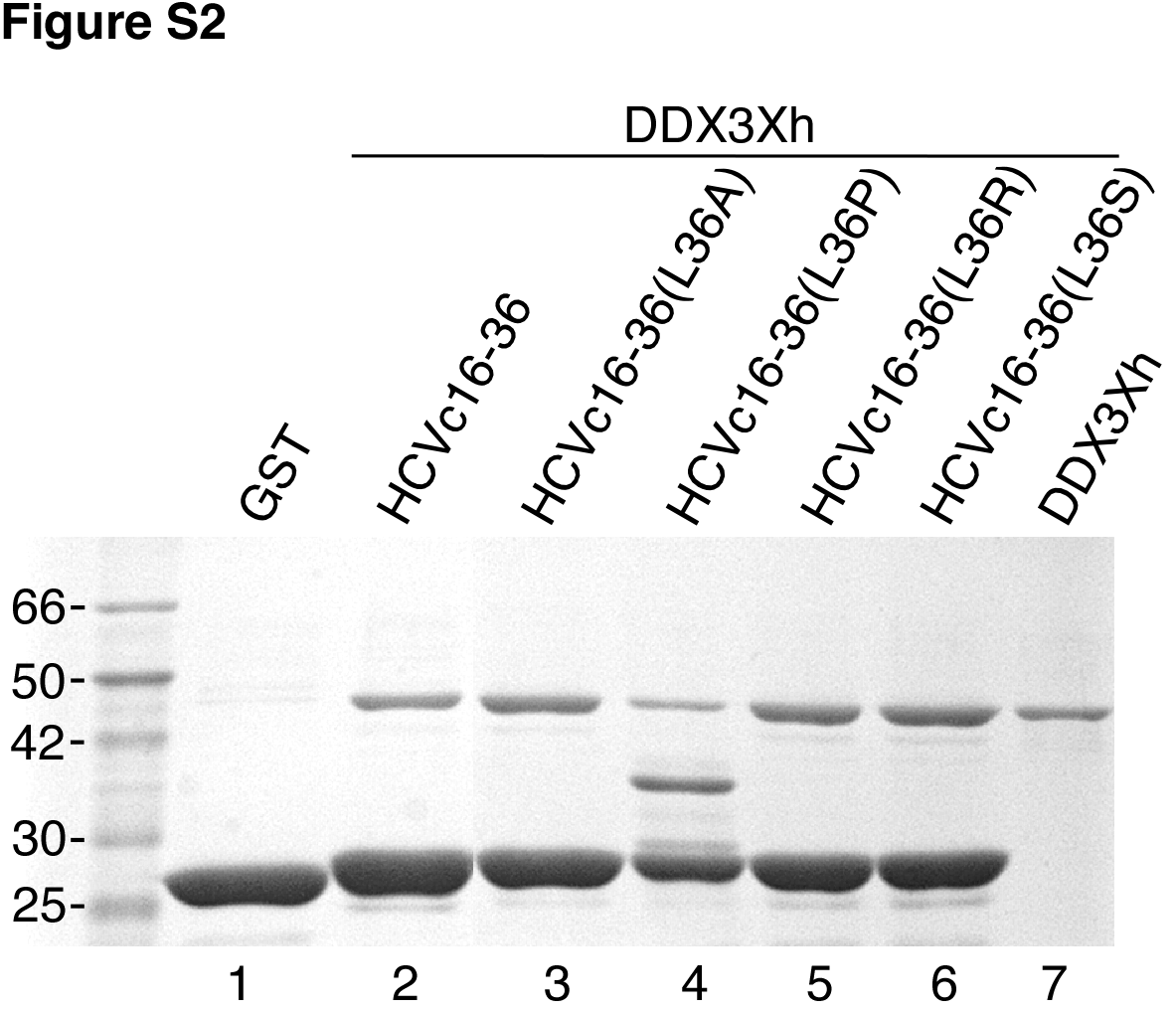

Supplement: Figure S2 — Pull-down assays of HCV core derived peptides with mutations at position 36 and DDX3X helicase domain (DDX3Xh). Analysis of the interaction between DDX3Xh, GST or various GST fusions to HCV core proteins mutated at position 36. Glutathione beads were used to pull down bound proteins prior to SDS gel electrophoresis. Lanes 2–6, GST fusions to the denoted HCV peptides incubated with DDX3Xh. Lanes 1 and 7, GST and DDX3Xh markers, respectively. Molecular weight markers are shown to the left, in kDa. (0.49 MB TIF) [file pone.0012826.s002.tif]

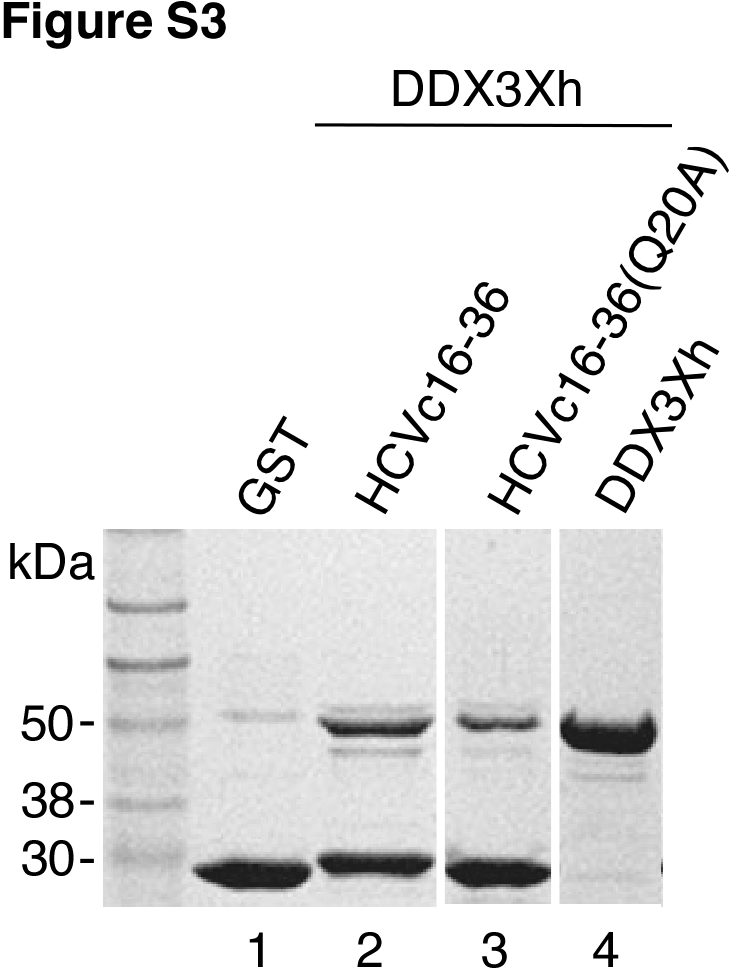

Supplement: Figure S3 — Pull-down assays of HCV core derived peptides mutated at position 20 and DDX3X helicase domain (DDX3Xh). Analysis of the interaction between DDX3Xh, GST or GST fusions to HCV core peptides. Glutathione beads were used to pull down bound proteins prior to SDS gel electrophoresis. Lanes 2–3, GST fusion to the denoted HCV peptides incubated with DDX3Xh. Lanes 1 and 4, GST and DDX3Xh markers, respectively. All samples were resolved on the same SDS gel, with intervening lanes removed for clarity. Molecular weight markers are shown to the left, in kDa. (0.18 MB TIF) [file pone.0012826.s003.tif]
